# Common Marker Genes Identified from Various Sample Types for Systemic Lupus Erythematosus

**DOI:** 10.1371/journal.pone.0156234

**Published:** 2016-06-03

**Authors:** Peng-Fei Bing, Wei Xia, Lan Wang, Yong-Hong Zhang, Shu-Feng Lei, Fei-Yan Deng

**Affiliations:** 1 Center for Genetic Epidemiology and Genomics, School of Public Health, Soochow University, Suzhou, Jiangsu 215123, P. R. China; 2 Jiangsu Key Laboratory of Preventive and Translational Medicine for Geriatric Diseases, School of Public Health, Soochow University, Suzhou, Jiangsu 215123, P. R. China; Institut National de la Santé et de la Recherche Médicale (INSERM), FRANCE

## Abstract

**Objective:**

Systemic lupus erythematosus (SLE) is a complex auto-immune disease. Gene expression studies have been conducted to identify SLE-related genes in various types of samples. It is unknown whether there are common marker genes significant for SLE but independent of sample types, which may have potentials for follow-up translational research. The aim of this study is to identify common marker genes across various sample types for SLE.

**Methods:**

Based on four public microarray gene expression datasets for SLE covering three representative types of blood-born samples (monocyte; peripheral blood mononuclear cell, PBMC; whole blood), we utilized three statistics (fold-change, FC; t-test p value; false discovery rate adjusted p value) to scrutinize genes simultaneously regulated with SLE across various sample types. For common marker genes, we conducted the Gene Ontology enrichment analysis and Protein-Protein Interaction analysis to gain insights into their functions.

**Results:**

We identified 10 common marker genes associated with SLE (IFI6, IFI27, IFI44L, OAS1, OAS2, EIF2AK2, PLSCR1, STAT1, RNASE2, and GSTO1). Significant up-regulation of IFI6, IFI27, and IFI44L with SLE was observed in all the studied sample types, though the FC was most striking in monocyte, compared with PBMC and whole blood (8.82–251.66 vs. 3.73–74.05 vs. 1.19–1.87). Eight of the above 10 genes, except RNASE2 and GSTO1, interact with each other and with known SLE susceptibility genes, participate in immune response, RNA and protein catabolism, and cell death.

**Conclusion:**

Our data suggest that there exist common marker genes across various sample types for SLE. The 10 common marker genes, identified herein, deserve follow-up studies to dissert their potentials as diagnostic or therapeutic markers to predict SLE or treatment response.

## Introduction

Systemic lupus erythematosus (SLE) is a chronic autoimmune disease with multiple organ involvement, in which auto-antibodies induce tissue damage[1]. The pathogenesis of SLE is complex, under the influence of both genetic and environmental factors. Recent genome-wide association studies (GWAS) identified a list of susceptibility genes for SLE [2–5], though their pathogenic mechanisms still await investigation. Besides, a number of gene expression studies on SLE cases and controls have been accumulated, leading to successful identification of gene expression signatures in various sample types, primarily in blood-born samples, including whole blood, peripheral blood mononuclear cells, monocyte, etc[6–9]. Previous such microarray gene expression studies usually focused on a specific type of SLE-related sample to identify genes regulated with SLE. So far, it is unknown whether there are commonly regulated genes with SLE among various sample types, which may contribute to future translational medicine in SLE prevention and treatment. Besides, it is unclear how genes identified from microarray expression study are related to genes independently identified by GWAS. The major purpose of this study is to identify significant marker genes which are commonly regulated in various sample types thus of general importance for SLE. In addition, we are also attempted to explore their interaction with known susceptibility genes reported by GWAS, so as to construct a network for all SLE-related genes identified by transcriptomics and genomics thus far. Through analyzing public microarray gene expression datasets, we successfully identified marker genes commonly regulated with SLE among various types of human blood-born samples.

## Materials and Methods

To find out whether there are common marker genes for SLE, we downloaded public microarray gene expression datasets generated from different types of SLE-related samples. Herein, we only focus on blood-born samples as blood sample is the most easily available, most widely used in clinical research, thus of greatest potential for future translational application. The data processing and analyses workflow of the present study was presented in S1 **Fig** and briefly introduced as follows.

### Identification of eligible gene expression datasets

We searched the NCBI PubMed and Gene Expression Omnibus (GEO) database (http://www.ncbi.nlm.nih.gov/geo/)[10] with key words “Systemic lupus erythematosus”, “SLE”, and “Gene expression profile”. A study was included in our analysis if it meets with the following three requirements: (1) it included both SLE patients and normal controls; (2) it contained gene expression profiling of blood-born samples, such as whole blood, peripheral blood mononuclear cells (PBMC), or monocytes; (3) the patients with immunosuppressive or prednisone medication or the samples treated with inducer were excluded. Finally, by April 13, 2015, four datasets were eligible and retained for subsequent analysis. Basic information for the four datasets and related samples, as published in the original articles and/or released with the datasets, were collected and presented in **Table 1**.

**Table 1:** Information of the Eligible Microarray Gene Expression Datasets.

GEO Accession	GSE46907	GSE50772	GSE61635	GSE49454
Platform	GPL96	GPL570	GPL570	GPL10558
Tissue	monocyte	^1^.PBMC	whole blood	whole blood
Ethnicity	NA	^2^^.^ Mixed	Mixed	Mixed
Number of Samples(case/control)	5/5	61/20	99/30	157/20
Number of Subjects(case/control)	5/5	61/20	79/30	62/20
Age(years), range	6–22	20~73	25–51	18–70
Female,%	>80%	>80%	92%	85%
SLEDAI, range/meadian	0-22/12	0~13/NA	0~31/6	0-22/NA
Diease duration(month), range/mean	0–24.7,8.3	NA	0~453,37.5	0~480,93.6
Reference	[6]	[7]	[8]	[9]

Note

^1.^ PBMC: peripheral blood mononuclear cells; SLEDAI: SLE disease activity index; NA: not available.

^2.^ Mixed: White, Black, plus Hispanic or Asian or others.

SLE cases and healthy controls were age- and gender- matched in the original studies.

Datasets were queried at http://www.ncbi.nlm.nih.gov/geo/query/acc.cgi.

### Data preprocessing

We downloaded the four eligible microarray gene expression datasets from GEO, and processed the datasets by using Microsoft Excel. As the four datasets were generated by three different experimental platforms, we firstly matched probe IDs to the unique official gene symbols, and then extracted the commonly profiled genes covered by all the four datasets. Finally, a total of 6,643 genes were retained and subject to further analysis.

### Multi-dataset analysis for identification of common marker genes

We conducted the multi-dataset analyses to identify common marker genes for SLE across various blood-born samples. As described previously[11], the multi-dataset analyses have two main analysis measures: 1) analyze each gene separately (marginal effects) and then compare across genes; 2) simultaneously analyze the effects of all genes (joint effects) in a single model. The analyses procedures are briefly introduced as follows.

#### a. Analysis of marginal effects

Three statistical methods, i.e., fold-change (FC) method[11], T-test[12], Benjamini & Hochbergfalse discovery rate (FDR) method[13], were used to analyze each dataset individually to identify significant genes for SLE in each type of sample, individually. Secondly, we ranked the FCs with decreasing order and T-test p values with increasing order within each dataset, and selected top 100 ranked genes in each dataset. Meanwhile, genes with significantFDR-adjusted-p values (FDR = 0.001) were selected. Thirdly, genes selected in at least three of the four studied datasets by eachof the above three methods were identified. Lastly, genes identified by the three methods were combined as a pool of common marker genes.

#### b. Analysis of joint effects

Different from the analysis of marginal effects, the joint effect analysis put all genes simultaneously into a model. It is expected that among large numbers of profiled genes, only a small subset is associated with the disease status. With such considerations, the Lasso penalized estimate method was used [14,15]. Details of this method have been introduced previously thus not elaborated herein[11]. In this study, Lasso penalized estimate was realized using R package “glmnet”.

### Functional annotation analysis

To gain insight into the biological functions of the common marker genes identified above, we performed Gene Ontology (GO) enrichment analysis (http://www.geneontology.org/). Herein, biological process was considered.

### Protein-Protein Interaction (PPI) analyses between common marker genes and GWAS-reported susceptibility gene

To explore the relationship between the common marker genes identified by transcriptomics and known SLE susceptibility genes identified by GWAS, we conducted PPI analysis for the two sets of genes by using the Search Tool for the Retrieval of Interacting Genes/Proteins (STRING) 10 Server (available at http://www.string-db.org/). At first, we collected GWAS-reported SLE susceptibility genes with p-value less than 1.0E-05(downloaded at http://www.genome.gov/gwastudies/index.cfm?pageid=26525384#searchForm). As of September 15^th^, 2015, a total of 91 such genes had been archived for SLE (**S1 Table).** Then, GWAS-reported genes, together with the common markers genes, were analyzed for PPI by the STRING.

## Results

### Study characteristics

Basic characteristics of the four GEO datasets (accession number: GSE61635, GSE50772, GSE46907 and GSE49454), which were utilized in the present study, were presented in **Table 1**. They were generated forboth SLE patients and healthy controls, with total RNA extracted from peripheral blood, peripheral blood mononuclear cells (PBMC), or monocytes, respectively. A total of 6643 genes, profiled in all the four datasets, were extracted and analyzed for both marginal and joint effects.

### Identification of common marker genes across the four datasets

Top 100 significant genes, selected by ranking with fold-change, were listed for each datasets in **S2 Table**. Among the list, 20 genes were repeatedly selected in at least three of the four studied datasets. Top 100 significant genes, selected by ranking with t-test p values, were listed for each datasets in **S3 Table**. Among the list, 10 genes were repeatedly selected in at least three datasets. Genes, selected with FDR 0.001, were listed for each datasets in **S4 Table**. Among the list, 121 genes were repeatedly selected from at least three of the four studied datasets. Genes, selected in at least three datasets, were combined for the three methods in **S5 Table**. Taken together, a total of 126 genes were identified as common genes by the three statistical methods. Most notably, two genes (IFI6 and OASL) were simultaneously selected by all the three methods (**S5 Table**, **S2A Fig**).

With the Lasso penalized estimate method, 56 genes were selected significant for SLE (**S6 Table)**.

Taken both marginal effect and joint effect analyses results together, a total of 172 genes were selected in at least three of the four studied datasets. Among the total 172 genes, 10 genes were simultaneously selected by both marginal and joint effect analyses (taken as common marker genes,**S2B Fig).** These overlapped genes are IFI6, IFI27, IFI44L, OAS1, OAS2, EIF2AK2, PLSCR1, STAT1, RNASE2, and GSTO1. Tracked back to marginal effect analyses results, as highlighted in **S5 Table**, among these 10 genes, one gene (IFI6) was selected by all the three marginal effect analyses methods; six genes (IFI27, IFI44L, OAS1, OAS2, EIF2AK2, and PLSCR1) were selected by two marginal effect analyses methods; and the remaining three genes (STAT1, RNASE2, and GSTO1) were solely selected by FDR method.

For the above 10 common marker genes, the fold-change and T-test and FDR-adjusted p-values in the original four studied datasets were presented in **Table 2**. All the 10 genes presented significant differential expression in both PBMC and whole blood samples.Three interferon-pathway genes (i.e., IFI6, IFI27, and IFI44L) showed consistently significantup-regulation in all the three studied sample types. Furthermore, fold-changesof the three interferon-pathway genes with SLE are much more striking in monocyte (FC range: 8.82–251.66), than in PBMC (FC range: 3.73–74.05) and whole blood (FC range: 1.19–1.87).

**Table 2:** Statistics (FC,T-test and FDR-adjusted p values) for the Ten Common Marker Genes in the Four Studied Datasets.

Gene symbol	GSE46907 (Monocyte)			GSE50772 (PBMC)			GSE61635 (Whole blood)			GSE49454 (Whole blood)		
	FC	p-value		FC	p-value		FC	p-value		FC	p-value	
		T-Test	FDR(0.001)		T-Test	FDR(0.001)		T-Test	FDR(0.001)		T-Test	FDR(0.001)
IFI6	**8.82**	**2.05E-03**	1.17E-05	**3.73**	5.36E-11	**3.67E-05**	**1.36**	**5.94E-28**	**1.34E-05**	**1.19**	**7.12E-17**	**2.86E-06**
OAS1	3.38	**2.85E-05**	1.51E-07	2.51	2.80E-08	**8.82E-05**	1.3	**8.94E-30**	**8.43E-06**	**1.24**	**5.33E-21**	**1.35E-06**
OAS2	**6.43**	**3.20E-04**	1.81E-06	2.08	6.14E-09	**7.11E-05**	1.22	**3.04E-29**	**1.02E-05**	**1.21**	**1.19E-15**	**3.16E-06**
EIF2AK2	3.08	**4.96E-04**	3.61E-06	2.04	1.70E-10	**4.34E-05**	1.12	**2.67E-29**	**9.94E-06**	**1.22**	**1.78E-11**	**5.27E-06**
PLSCR1	2.36	**5.45E-05**	1.05E-06	2.02	3.36E-12	**2.35E-05**	1.22	**9.46E-35**	**3.01E-06**	**1.19**	**2.58E-13**	**4.37E-06**
IFI27	**251.66**	6.78E-02	1.78E-04	**74.06**	1.14E-07	**1.04E-04**	**1.4**	6.06E-16	**1.25E-04**	**1.87**	**3.55E-18**	**2.41E-06**
IFI44L	**11.7**	1.07E-03	6.62E-06	**5.55**	4.72E-10	**5.04E-05**	**1.35**	5.55E-22	**4.43E-05**	**1.52**	**1.24E-24**	**4.52E-07**
STAT1	2.68	4.38E-03	2.32E-05	1.63	5.11E-09	**6.82E-05**	1.07	8.85E-17	**1.10E-04**	1.1	**6.93E-15**	**3.31E-06**
RNASE2	3.8	1.87E-02	7.21E-05	**3**	1.53E-11	**2.98E-05**	1.06	2.67E-06	**4.35E-04**	**1.17**	**4.63E-10**	**7.83E-06**
GSTO1	1.02	0.889	9.31E-04	1.65	**3.10E-15**	**5.87E-06**	1.01	5.76E-04	**5.81E-04**	1.04	6.52E-06	**3.01E-05**

Note: FC: fold-change; FDR: false discovery rate. Values **in bold** mean the corresponding genes were selected as significant from the corresponding datasets/samples under the corresponding methods. IFI6: interferon, alpha-inducible protein 6; OAS1: 2'-5'-oligoadenylate synthetase 1; OAS2: 2'-5'-oligoadenylate synthetase 2; EIF2AK2: eukaryotic translation initiation factor 2-alpha kinase 2; PLSCR1: phospholipid scramblase 1; IFI27:interferon, alpha-inducible protein 27; IFI44L:interferon-induced protein 44-like; STAT1:signal transducer and activator of transcription 1; GSTO1:glutathione S-transferase omega 1; RNASE2:ribonuclease, RNase A family, 2.

### Functional annotation analysis

The top significant enriched biological processes, for the 10 common genes identified above for SLE, were shown in **Table 3**. The most significantly enriched functions were “response to virus” (GO: 0009615, p = 1.7E-03) and “immune response” (GO: 0006955, p = 6.1E-03). Interestingly, they are also highly enriched in “RNA catabolic process”, “regulation of enzyme activity including caspase, endopeptidase, and peptidase activity”, and “apoptosis and programmed cell death”.

**Table 3:** The Significantly Enriched GO Terms of Biological Processes for the Ten Common Marker Genes Identified.

GO Term	Biological Process	P-value	Marker Gene
GO:0009615	response to virus	1.75E-03	PLSCR1,EIF2AK2,STAT1
GO:0006955	immune response	6.10E-03	IFI44L,OAS1,OAS2,IFI6
GO:0006401	RNA catabolic process	3.78E-02	RNASE2,OAS2
GO:0043281	regulation of caspase activity	4.58E-02	STAT1,IFI6
GO:0052548	regulation of endopeptidase activity	4.75E-02	STAT1,IFI6
GO:0052547	regulation of peptidase activity	4.98E-02	STAT1,IFI6
GO:0006915	apoptosis	4.63E-02	EIF2AK2,STAT1,IFI6
GO:0012501	programmed cell death	4.76E-02	EIF2AK2,STAT1,IFI6

Note: The GO analysis was performed in the GO web site (http://www.geneontology.org/).

### PPI analyses between common marker genes and GWAS-reported gene

PPI analysis showed complex interactions for the 10 common marker genes with known SLE susceptibility genes.

**Fig 1:**
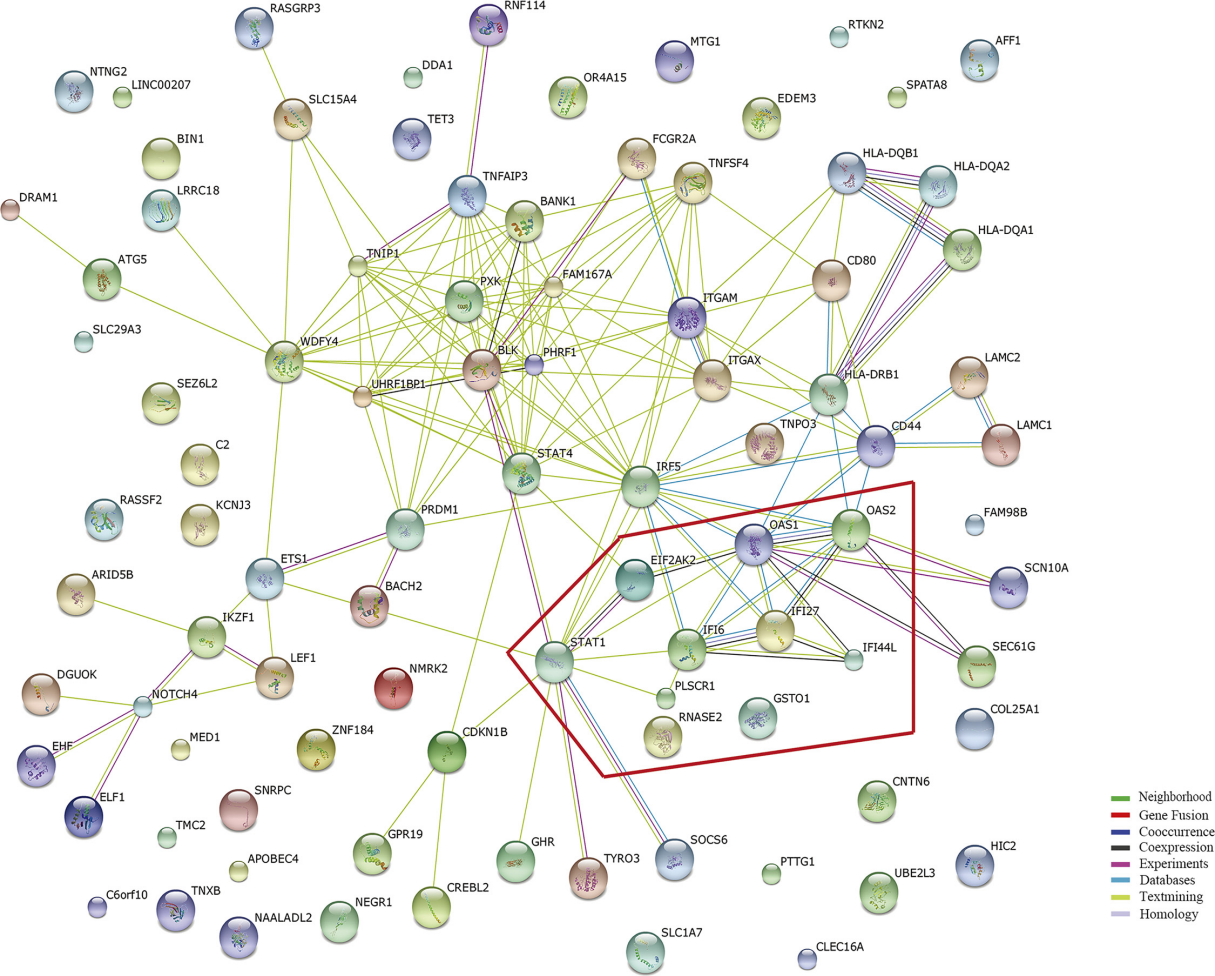
The Evidence View of Protein-Protein Interaction. The PPI were analyzed using the STRING database 10. The predicted functional interaction network is shown in the evidence view where the different line colors represent different types of evidences for the association. The nodes inside the red polygon represent the common genes we identified in the present study.

On one hand, there exist evidences supporting internal interactions within the common marker genes (**Fig 1**). For example, text mining evidences support that IFI6 has connection with the other common genes, including STAT1, PLSCR1, IFI44L, IFI27, OAS2, and OAS1. In addition, STAT1 interacts with EIF2AK2, and the latter co-expresses with OAS1. Experimental evidence supports OAS1 and OAS2 interact with each other. Besides, OAS1 co-expresses with IFI44L, whereas OAS2 co-expresses with IFI27. Both IFI27 and IFI44L co-express with IFI6.

On the other hand, there exist evidences supporting that all the above identified common marker genes,except RNASE2 and GSTO1 genes, have direct or indirect interactions with GWAS-reported SLE susceptibility genes (**Fig 1, S7 Table**). For example, STAT1 shows text-mining interactions with IRF5, ITGAX, ITGAM, BLK, ETS1, CDKN1B, GHR, TYRO3, and SOCS6. Among those interactions, interactions forSTAT1 with BLK, TYRO3, and SOCS6 have been further supported by experimental evidences. Besides, experimental evidence also supports interaction for the two homolog genes, OAS1 and OAS2, with SEC61G.

To be noted, among the 10 common marker genes, RNASE2 and GSTO1 were selected with statistical evidences as strong as STAT1. As described above, lots of evidences support interaction for STAT1 withother marker genes or susceptibility genes for SLE. In contrast, no interaction evidences have been obtained thus far supporting that RNASE2 and GSTO1 interact with GWAS-reported genes or other common marker genes.

## Discussion

SLE is mainly characterized by auto-antibodies-induced tissue damage. Abnormal immune process and inflammation are important pathologic processes for SLE. Previous GWAS and microarray gene expression studies have identified a large number of genes for SLE, which improved our understanding of the genetic pathogenesis of SLE. As various sample types have been utilized in gene expression microarray studies, it would be interesting to evaluate which genes are of general significance for SLE from translational medicinepoint of view. In this study, we were attempted to identify commonly regulated genes with SLE in various sample types, which may have potentials for translational application for SLE. In the present study, based on four public microarray gene expression datasets with case-control study design, we have dug out 10 significant genes (IFI6, IFI27, IFI44L, OAS1, OAS2, EIF2AK2, PLSCR1, STAT1, RNASE2, and GSTO1) which are commonly regulated with SLE in three representative types of blood-born samples.

It is well known that immune response and tissue damage areinvolved in the development of SLE. Our GO analyses showed that the 10 common marker genes, identified in this study, not only interact with each other but also interact with GWAS-reported genes, participate in immune response, regulate RNA catabolism and protein degradation, and play significant roles in programmed cell death and apoptosis. These biological functions areconsistent with the pathologic characteristics of SLE, indicating that thesegenes are fundamental to SLE pathogenesis.

From the expression pattern point of view, all the 10 genes presented significant differential expression in both PBMC and whole blood between SLE patients and controls. Notably, consistently significant up-regulation of three interferon-pathway genes (i.e., IFI6, IFI27, and IFI44L) with SLE was observed in all the three studied sample types, including monocyte. Meanwhile, monocyte sample presents a most striking fold change for these three genes, as compared with PBMC and whole blood (8.82–251.66 vs. 3.73–74.05 vs. 1.19–1.87), implying thatmonocyte is a dominant, if not exclusive, cell type actively expressing IFI6, IFI27, and IFI44Lin SLE patients’whole blood.The above observations suggest that monocyte-expressed interferon pathway genes are significantly involved in the pathogenesis of SLE.

Consistently, evidences from previous studies support that seven of the above 10 common genes were relevant to SLEpathogenesis and therapeutics. For example, EIF2AK2 [16], IFI27 [16,17], OAS1[18–23], OAS2[21,23,24], PLSCR1[16] and STAT1[25–27] were up-regulated in SLE patients vs. controls.Specifically, up-regulation of IFI27 and IFI44L in SLE patients was further observed in the synovial tissue[26]. Besides, OAS1 was also associated with SLE disease activity [18], whereas OAS2 expression was positively correlated with expression level of Syk, which is a potential therapeutic target of SLE [28]. Furthermore, STAT1 may regulate expression of type I interferon, which was related to SLE susceptibility [29]. In addition, STAT1 may indicate therapeutic action in SLE patients[30].

In general, the above evidences warrant the importance of the identified common marker genes for SLE.

Besides the above seven genes previously recognized to be relevant to SLE, the present study firstly points out that IFI6,RNASE2, and GSTO1 genes are novelmarker genes for SLE. To be noted, significance of IFI6 and RNASE2 to another kind of auto-immune disease, i.e., rheumatic arthritis (RA), has been reported. IFI6 was a candidate biomarker predictive of therapeutic responses to tocilizumab in patients with RA [31]. RNASE2 was found significantly up-regulated in RA patients vs. controls [32,33]. Taken all related evidences together, the three genes deserve more attention for investigation. Specifically, whether IFI6 would be predictive of therapeutic responses to tocilizumabin patients with SLE remains an interesting clinical question, which has yet to be answered by further research.

The present study is purposed to identify common marker genes shared in various SLE-related human sample types through mining deeper into public microarray expression datasets. Due to limited data resources, we only focused on three representative types of blood-born samples in this study. Consequently, this study is not comprehensive in incorporating all kinds of SLE-related samples, e.g., T cells. However, significant genes, identified in the present study, may serve as a pool of candidate ubiquitous marker genes for further exploration and validation in additional SLE-related sample types, which would become feasible when more and more gene expression dataset are being accumulated and archived in the near future. For example, among the ten marker genes commonly regulated in human blood-born samples, up-regulation of IFI27 and IFI44L in SLE patients has been validated in the synovial tissue [26], as well. This piece of evidence further highlights the significance of these two marker genes for SLE.

**Fig 2:**
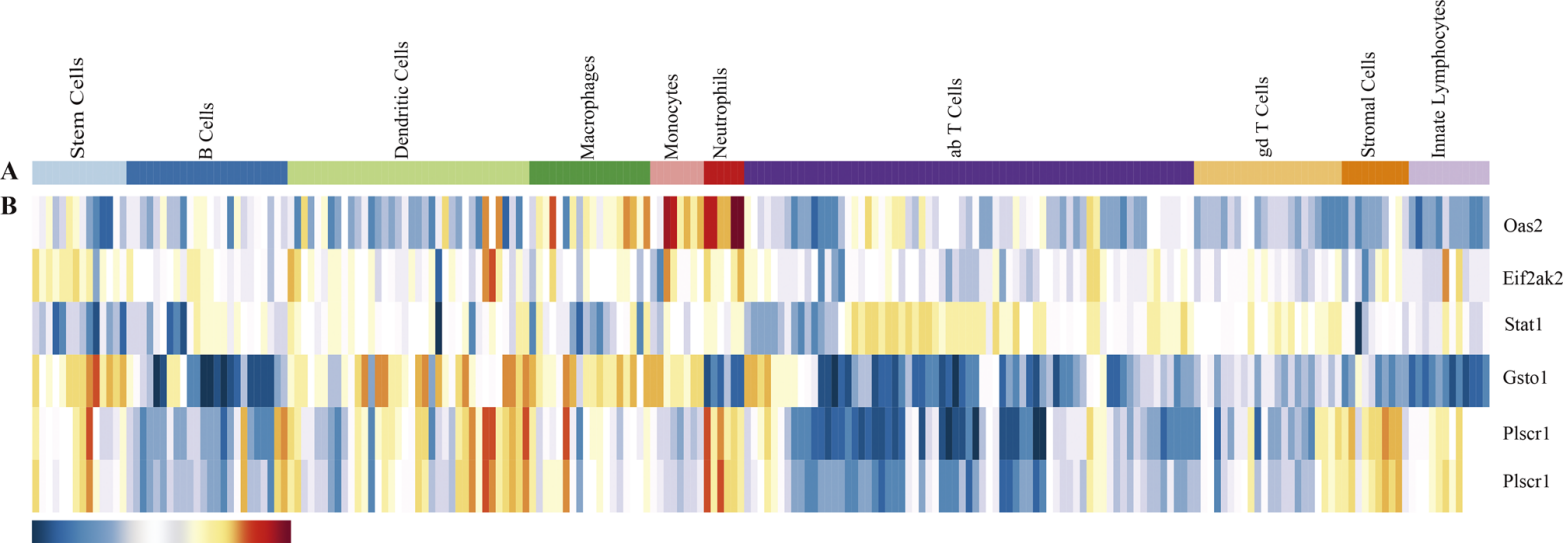
Overview of Gene Expression for the Common Marker Genes in Various Immune Cell Subsets. (A)The different bar colors represent different immune cell subsets. (B) The bar under the heatmap illustrates that the expression level increases gradually with the color changing from blue to red. The gene expression data was extracted from the ImmGen (available at http://www.immgen.org/). Two datasets were included in the ImmGen for the PLSCR1 gene. The Fig was processed on Dec 9, 2015.

To understand which immune cell subsets are contributing to the whole blood expression of the identified genes, we searched the Immunological Genome (ImmGen, available at http://rstats.immgen.org/MyGeneSet/) [34], which is a 'road map' of gene-expressionacross all immunecell populations. The gene expression pattern for five of the total 10 genes, across various human immune cell subsets, was available and presented in **Fig 2**. We can see that, thefive genes have various expression patterns. Among all immune cell subsets, OAS2,EIF2AK2, STAT1, and GSTO1 genes are most highly expressed in Neutrophils, Dendritic Cells, T Cells, and Stem Cells, respectively. PLSCR1 is mostly highly expressed in Neutrophils, Dendritic Cells, and Macrophages.This referenceexpression pattern, together with expression patternunder disease status, may be helpful for ascertaining key immunological cell subsets for a specific immune diseases, including SLE.

The present study identified 10 common markers genes for SLE in three types of blood-born samples. Based on their statistical signficance demonstrated in the present study andtheir relevance to SLEdiscussed as above, these mRNA markers deserve follow-up studies 1) to investigate their functional mechanisms, regarding how they are invovled in SLE pathogenesis, for example, how monocyte-expressed interferon pathway genes are involved in the pathogenesis of SLE; 2) to investigate their values in early prevention, regarding whether they can serve as diagnostic markers to predict SLE; 3) to investigate their values in disease treatment, regarding whether they can serve as markers predictive of therapeutic responses in SLE patients.

In conclusion, this study suggests that there are commonly regulated genes in various sample types for SLE. We identified 10 common marker genes, which are cross-validated in multiple types of human blood-born samples. In-depth cellular functional studies may improve our understanding of pathogenesis for SLE. Meanwhile, follow-up studies are needed to dissert their potentials as diagnostic or therapeutic markers to predict SLE or predict treatment response.

## Supporting Information

S1 Fig Flowchart of Data Processing and Data Analyses. (DOCX)

S2 Fig The Venn Diagram of Genes Identified by Marginal Effect Analyses (A), and by both Marginal and Joint Effect Analyses (B).
(DOCX)

S1 Table Known Susceptibility Genes for SLE reported by GWAS. (XLSX)

S2 Table Top 100 Significant Genes Selected from Each Dataset by Fold-Change. (XLSX)

S3 Table Top 100 Significant Genes Selected from Each Dataset by T-test P Value. (XLSX)

S4 Table Significant Genes Selected by FDR-adjusted P Value (FDR = 0.001). (XLSX)

S5 Table Genes Selected from ≧3 Datasets by each Marginal Effect Analysis Method. (XLSX)

S6 Table Genes Identified by the Lasso Penalized Estimate. (XLSX)

S7 Table Interactions between the Common Marker Genes Identified in the Present Study and Known Susceptibility Genes Reported by GWAS for SLE. (DOCX)
