# Supplementary material for: Common Marker Genes Identified from Various Sample Types for Systemic Lupus Erythematosus
Source: PLoS One. 2016 Jun 3;11(6):e0156234. doi: 10.1371/journal.pone.0156234 (PMC4892593; doi:10.1371/journal.pone.0156234)
Supplement: S1 Fig — (DOCX) [file pone.0156234.s001.docx]

Four datasets downloaded from GEO

Common genes extracted (6643)

FC top 100 (20 genes)

T test top 100 (10 genes)

Marginal effect (126)

Joint effect (56)

Lasso penalized estimate (56)

FDR0.001 (121 genes)

GWAS-reported susceptibility genes (91)

Overlapped genes (10)

PPI analysis

GO analysis

**S1 Fig. Flowchart of Data Processing and Data Analyses**

Note: the numbers in brackets are numbers of genes selected by the corresponding analysis methods.
