# Supplementary material for: Common Marker Genes Identified from Various Sample Types for Systemic Lupus Erythematosus
Source: PLoS One. 2016 Jun 3;11(6):e0156234. doi: 10.1371/journal.pone.0156234 (PMC4892593; doi:10.1371/journal.pone.0156234)
Supplement: S2 Fig — The Venn Diagram of Genes Identified by Marginal Effect Analyses (A), and by both Marginal and Joint Effect Analyses (B). (DOCX) [file pone.0156234.s002.docx]

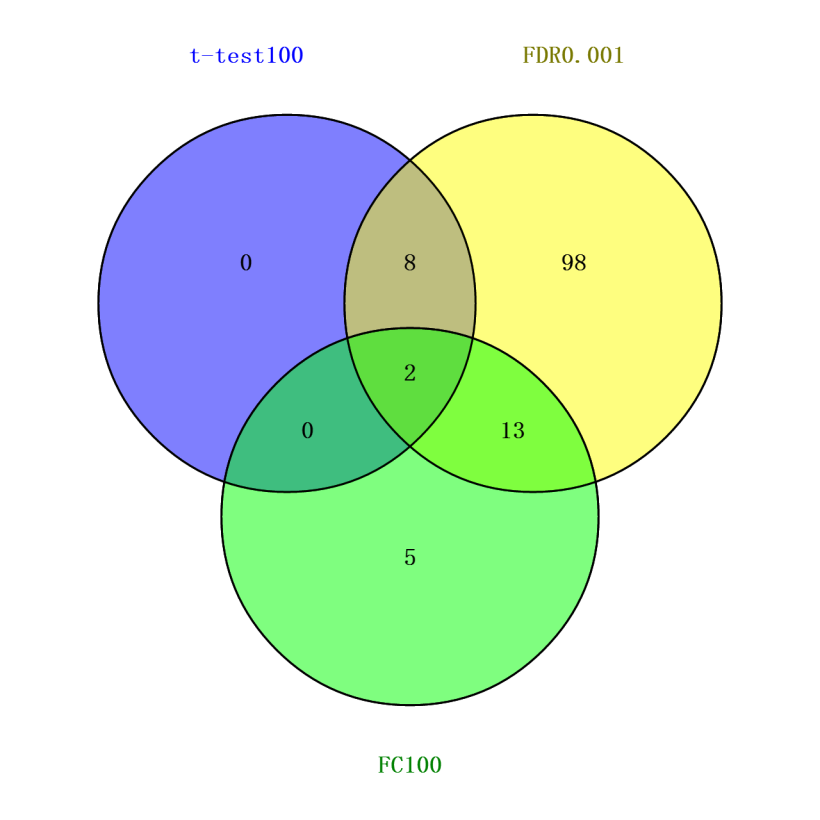

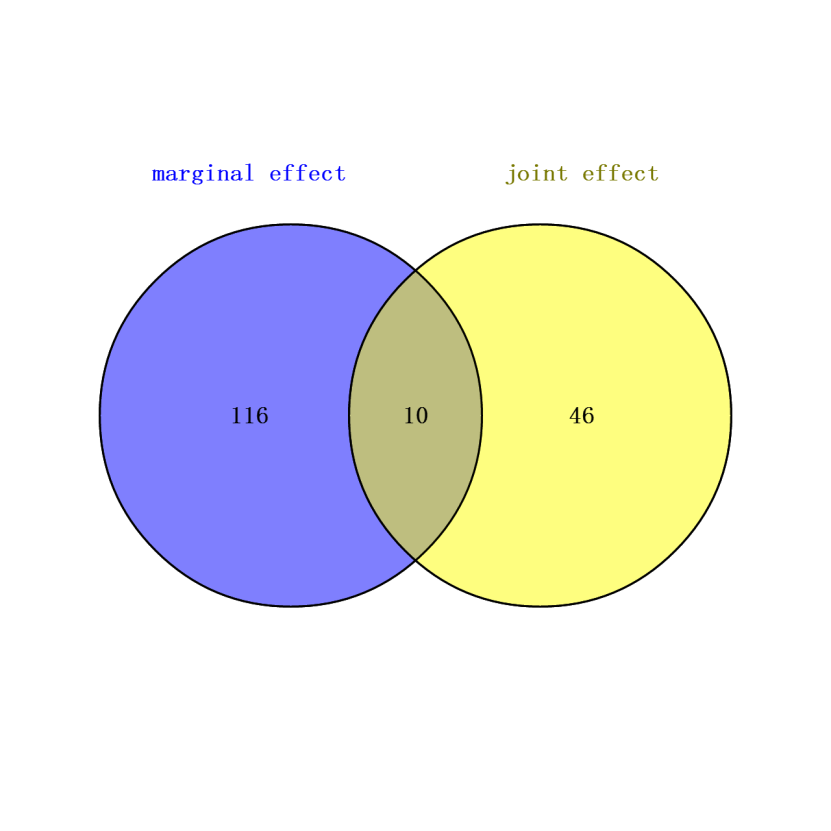


**S2 Fig. the Venn Diagram of Genes Identified by Marginal Effect Analyses (A), and by both Marginal and Joint Effect Analyses (B).**
