# Supplementary material for: Common Marker Genes Identified from Various Sample Types for Systemic Lupus Erythematosus
Source: PLoS One. 2016 Jun 3;11(6):e0156234. doi: 10.1371/journal.pone.0156234 (PMC4892593; doi:10.1371/journal.pone.0156234)
Supplement: S7 Table — (DOCX) [file pone.0156234.s009.docx]

**S7 Table．Interactions between the Common Marker Genes Identified in the Present Study and Known Susceptibility Genes Reported by GWAS for SLE**

| Marker genes | Conjoint genes | GWAS-P-value (Catalog) | Connection type |
| --- | --- | --- | --- |
| **IFI6** | IRF5 | 7.00E-10 | D/T |
|  | STAT1 | — | T |
|  | PLSCR1 | — | T |
|  | IFI44L | — | C2/T |
|  | IFI27 | — | D/T/H |
|  | OAS2 | — | D/T |
|  | OAS1 | — | D/T |
| **OAS1** | IRF5 | 7.00E-10 | D |
|  | EIF2AK2 | — | C2/T |
|  | STAT1 | — | T |
|  | IFI6 | — | D/T |
|  | IFI27 | — | D/T |
|  | IFI44L | — | C2/T |
|  | SEC61G | 2.00E-06 | C1/E |
|  | OAS2 | — | C1/E/T/H |
|  | CD44 | 2.00E-07 | D/T |
|  | HLA-DRB1 | 3.00E-07 | D |
| **OAS2** | OAS1 | — | C1/E/T/H |
|  | IFI6 | — | D/T |
|  | IFI27 | — | C1/D/T |
|  | SEC61G | 2.00E-06 | C1/E |
|  | SCN10A | 7.00E-06 | G/T |
|  | CD44 | 2.00E-07 | D |
|  | HLA-DRB1 | 3.00E-07 | D |
| **EIF2AK2** | STAT4 | 5.00E-09 | T |
|  | STAT1 | — | G/C2/T |
|  | OAS1 | — | C2/T |
| **PLSCR1** | IFI6 | — | T |
|  | STAT1 | — | T |
| **IFI27** | IRF5 | 7.00E-10 | D/T |
|  | IFI6 | — | D/T/H |
|  | IFI44L | — | C1/T |
|  | OAS2 | — | D/T/H |
|  | OAS1 | — | D/T |
| **IFI44L** | OAS1 | — | C2/T |
|  | IFI6 | — | C2/T |
|  | IFI27 | — | C2/T |
| **STAT1** | IRF5 | 7.00E-10 | T |
|  | ITGAX | 3.00E-11 | T |
|  | ITGAM | 2.00E-06 | T |
|  | BLK | 2.00E-08 | E/T |
|  | ETS1 | 3.00E-08 | T |
|  | CDKN1B | 5.00E-12 | T |
|  | GHR | 4.00E-06 | T |
|  | TYRO3 | 1.00E-14 | E/T |
|  | SOCS6 | 6.00E-06 | N/E/T |
|  | PLSCR1 | — | C2/T |
|  | IFI6 | — | T |
|  | OAS1 | — | T |
|  | EIF2AK2 | — | C1/E/T |

Note: The 10 common marker genes, except RNASE2 and GSTO1, were found to have connection with each other and with the GWAS-reported genes. The minimum p-values reported by GWAS for each SLE susceptibility gene were presented in the Table as GWAS-P-value (Catalog). The connection types N, G, C1, C2, E, D, T and H represent Neighborhood, Gene Fusion, Cooccurrence, Coexpression, Experiment, Database, Textmining, and Homology. The transverse line “-” means that the interacted genes are common marker genes identified in the present study but not reported by GWAS.
